# Association between visceral obesity and 10-year risk of first atherosclerotic cardiovascular diseases events among American adults: National Health and Nutrition Examination Survey

**DOI:** 10.3389/fcvm.2023.1249401

**Published:** 2023-08-21

**Authors:** Liying Zheng, Aochuan Sun, Senfu Han, Rongming Qi, Rumeng Wang, Xiao Gong, Mei Xue

**Affiliations:** ^1^ National Clinical Research Center for Chinese Medicine Cardiology, Xiyuan Hospital, China Academy of Chinese Medical Sciences, Beijing, China; ^2^ Xiyuan Hospital, China Academy of Chinese Medical Sciences, Beijing, China; ^3^ Graduate School, Beijing University of Chinese Medicine, Beijing, China

**Keywords:** atherosclerotic cardiovascular disease, visceral obesity, visceral obesity index, lipid accumulation product, NHANES

## Abstract

**Background:**

In the United States, the relationship between visceral obesity and the risk of developing atherosclerosis cardiovascular disease (ASCVD) for the first time in 10 years is unclear.

**Methods:**

Data for this cross-sectional study came from the National Health and Nutrition Examination Survey (NHANES) from 2011 to 2020. We collected variable information related to 10-year ASCVD risk and visceral obesity reliable indicators [Visceral obesity index (VAI) and Lipid accumulation product (LAP)]. And we used multiple logistic regression to analyze the correlation of visceral obesity indicators (VAI and LAP) with 10-year ASCVD risk. In addition, we assessed the linear relationship between VAI or LAP and 10-year ASCVD risk by smoothing curve fitting. Finally, we conducted subgroup analysis and sensitivity analysis after excluding participants with extreme VAI and LAP values to ensure that we obtained accurate and reliable results.

**Results:**

Our study included a total of 1,547 participants (mean age: 56.5 ± 10.1, 60% of males). The results of the multiple logistic regression showed that compared with participants with the lowest VAI in the 1st Quartile (≤0.79), the adjusted OR values for VAI and elevated 10-year ASCVD risk in Q3 (1.30–2.14), and Q4 (≥2.15) were 2.58 (95% CI: 1.24–5.36, *P* = 0.011), 15.14 (95% CI: 6.93–33.05, *P* < 0.001), respectively. Compared with participants with the lowest LAP in the 1st Quartile (≤28.29), the adjusted OR values for VAI and elevated 10-year ASCVD risk in Q3 (46.52–77.00), and Q4 (≥77.01) were 4.63 (95% CI: 2.18–9.82, *P* < 0.001), 16.94 (95% CI: 6.74–42.57, *P* < 0.001), respectively. Stratified analysis showed that the association between VAI or LAP and the first ASCVD event was more pronounced in males.

**Conclusion:**

Higher VAI or LAP scores are significantly associated with elevated 10-year ASCVD risk in adults aged 40 to 79 in the USA, which suggested that monitoring visceral obesity is crucial to reduce the risk of a first ASCVD event.

## 1. Introduction

Despite encouraging achievements in the prevention, diagnosis, and treatment of ASCVD in recent years, ASCVD remains a leading cause of disability and premature death worldwide (1, 2). Due to the fact that most patients with early-onset ASCVD have modifiable risk factors before onset (3), exploring risk-related indicators for ASCVD and conducting early intervention is of paramount importance in reducing ASCVD mortality and alleviating the healthcare burden.

Obesity is a key risk factor for ASCVD (4–6), and the dramatic increase in obesity prevalence in recent years has undermined the gains made in controlling ASCVD risk factors and advancing medical technology (7). Body Mass Index (BMI) is a widely recognized standard for measuring obesity (8, 9). However, BMI can not distinguish between lean fat and whole fat, nor does it reflect the distribution of abdominal fat and body fat, and therefore it has some limitations in estimating the risk of ASCVD (10–12). Epidemiological findings suggested that visceral fat measured by imaging techniques such as CT or MRI is an independent risk factor for cardiovascular metabolic diseases and death (13). And there is evidence that ectopic fat deposition may be related to atherosclerosis and the increased risk of cardiometabolic (14). However, using techniques such as CT or MRI to measure ectopic fat deposition is expensive and limited by the detection instrument (15, 16). Therefore, several simple clinical tools have been developed to assess changes in visceral fat and ectopic fat deposition, of which VAI and LAP have been widely accepted and used clinically as two reliable indicators for assessing visceral obesity. VAI is a simple and reliable indicator of visceral adiposity dysfunction that reflects cardiometabolic risk, and is calculated by anthropometric parameters [waist circumference (WC) and BMI] and lipid measurement parameters [triglycerides (TG) and high-density lipoprotein cholesterol (HDL-C)] (17–19). LAP is an index of lipid hyperaccumulation based on WC and TG, and is considered to be a good continuous indicator to describe visceral obesity (20–22).

To our knowledge, few studies have been conducted on the association between visceral obesity and 10-year ASCVD risk, and the relationship between the two remains controversial. Therefore, we analyzed the association of VAI and LAP with 10-year ASCVD risk through a cross-sectional study to provide a scientific basis for clinical application.

## 2. Materials and methods

### 2.1. Study design and population

The National Health and Nutrition Examination Survey (NHANES) aims to assess and track the health and nutritional status of the non-institutionalized population in the United States through comprehensive health-related studies. A face-to-face interview is conducted at the individual's home to obtain information on demographics and medical history. Data from examinations, which include physiological, laboratory, and anthropometric data, were collected at the Mobile Examination Center (MEC). The NHANES protocol obtained approval from the National Center for Health Statistics ethics review committee and received written informed consent from all participants (23). For this cross-sectional study, we merged the NHANES data from 2011 to 2012, 2013–2014, 2015–2016, and 2017–2020. Participants included in this study had to meet the following criteria: (1) age between 40 and 79 years old, (2) no existing diagnosis of ASCVD, (3) HDL-C between 20 and 100 mg/dl, (4) total cholesterol (TC) between 130 and 320 mg/dl, and (5) systolic blood pressure (SBP) between 90 and 200 mmHg.

### 2.2. Measurement of VAI and LAP

The VAI and LAP was used as exposure variable and was calculated using gender-specific equations, as detailed below. VAI: male [WC/39.68 + (1.88 × BMI)] × (TG/1.03) × (1.31/HDL-C); female [WC/36.58 + 1.89 × (BMI)] × (TG/0.81) × (1.52/HDL-C) (24). LAP: male [WC – 65] × TG; female [WC – 58] × TG (25). TG (mmol/L) was measured using the Wahlefeld method and HDL-C (mmol/L) was measured using the magnesium sulfate/glucan method. The calculation method for BMI is to divide weight (kilograms, kg) by height (meters, m) squared (kg/m^2^). WC (cm) was measured with an accuracy of millimeters using electronic sports measurements.

### 2.3. ASCVD risk definition and assessment

The Pooled Cohort Equations (PCE) were implemented in 2013 by the American College of Cardiology/American Heart Association (ACC/AHA) as a tool for estimating the likelihood of developing ASCVD over ten years. This risk prediction model specifically caters to individuals aged 40–79 who are non-Hispanic white. This risk assessment equation includes characteristics such as age, gender, race, SBP, diastolic blood pressure (DBP), TC, HDL-C, low-density lipoprotein cholesterol (LDL-C), smoking status, hypertension treatment, statin use, and aspirin therapy. The 10-year risk of a first hard ASCVD event can be estimated by https://tools.acc.org/ASCVD-Risk-Estimator-Plus/#!/calculate/estimate/. Participants who scored ≥7.5% were classified as having an elevated 10-year ASCVD risk, whereas those who scored <7.5% were identified as low-risk individuals (26).

### 2.4. Statistical analysis

Continuous variables with normal distribution were expressed as mean ± standard deviation (SD), while those with skewed distribution were expressed as the median [interquartile range, (IQR)]. Categorical variables were presented as frequencies (%). The baseline characteristics of different 10-year ASCVD risk groups were compared using One-Way ANOVA when the data were normally distributed, Kruskal-Wallis H when the distribution was skewed, and the chi-square test for categorical variables analysis. We used logistic regression to investigate the association between VAI and LAP with 10-year ASCVD risk (odds ratios [OR] and 95% confidence interval [CI]). Both non-adjusted and multivariate adjusted models were utilized in this study. Model 1 included adjustments for age, gender, and race. Model 2 was adjusted for sociodemographic characteristics such as age, gender, race, education level, marital status, PIR, smoking status, and BMI. Model 3 encompassed full adjustments, including sociodemographic characteristics, blood pressure measurements (SBP and DBP), TC, LDL-C, diabetes, statin use, and aspirin therapy.

Furthermore, we employed a smoothed curve fitting approach to evaluate the linear association between VAI or LAP and 10-year ASCVD risk. To ensure the accuracy of the findings from this study, multivariate logistic regression models were used for subgroup analysis. Possible variations in the relationship between VAI or LAP and 10-year ASCVD risk were examined, including gender, race, diabetes, statin use, and aspirin therapy. The interaction between subgroups was assessed using the likelihood ratio test. Moreover, participants with extreme VAI and LAP outside the mean ± 3 SD were excluded, for sensitivity analyses. All statistical analyses were conducted utilizing R version 3.3.2 (The R Foundation, http://www.R-project.org) and Free Statistics software version 1.7). A two-sided *P* value <0.05 was regarded as having statistical significance.

## 3. Results

### 3.1. Study population

**Figure 1:**
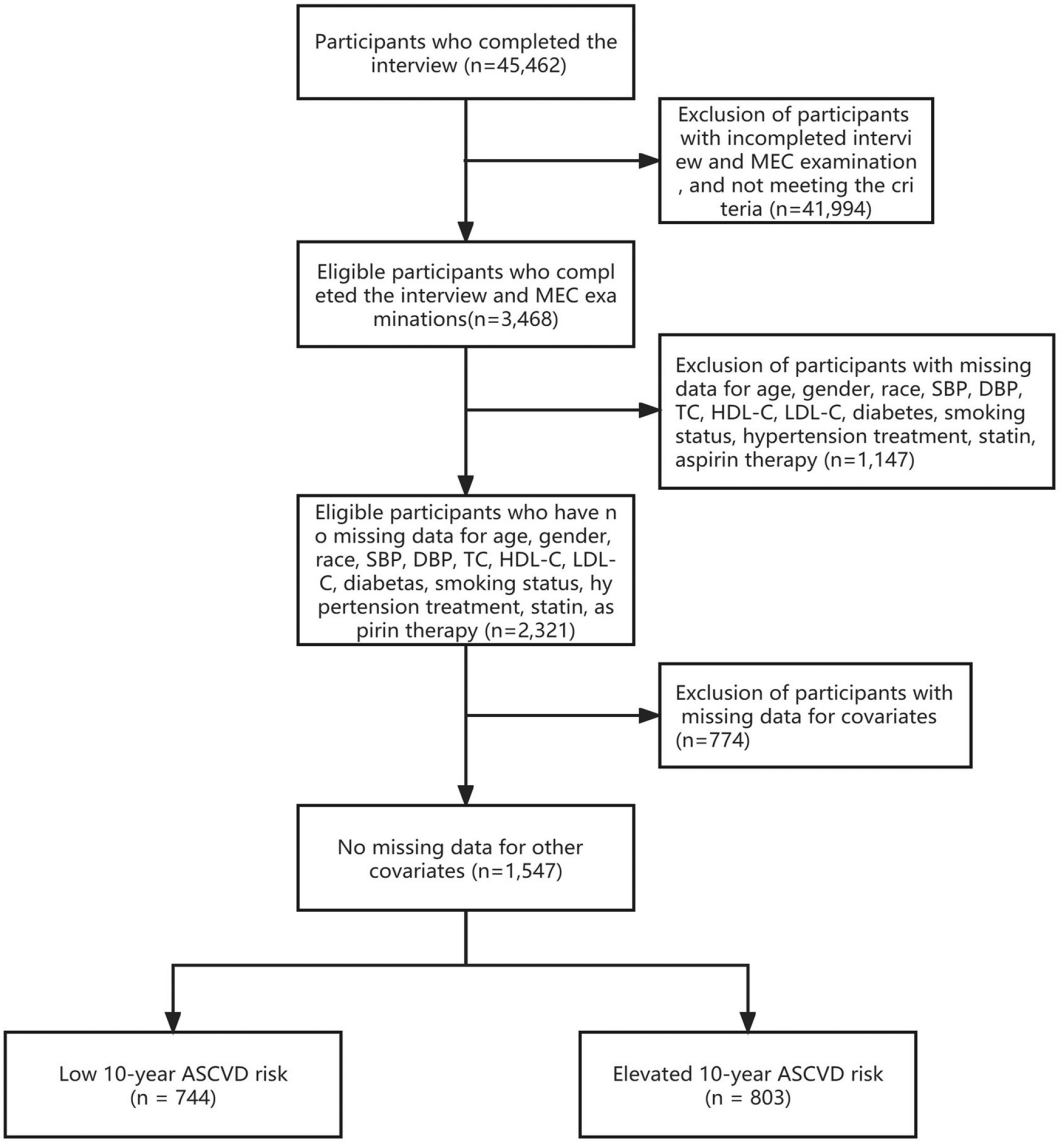
Flowchart of study population screening.

This study included 45,462 prospective participants from NHANES (2011–2020), of which 3,468 adults (40–79 years) who met the inclusion criteria completed interviews and were subjected to MEC screening. Participants with missing data for age, gender, race, SBP, DBP, TC, HDL-C, LDL-C, diabetes, smoking status, hypertension treatment, statin, and aspirin therapy were excluded (*n* = 1,147). After excluding participants with incomplete covariate data (*n* = 774), a total of 1,547 participants were enrolled in this cross-sectional study. The flowchart of population screening is shown in Figure 1.

### 3.2. Characteristics of participants

The mean participants' age was 56.5 ± 10.1 years, and 928 (60.0%) were men. The mean baseline VAI and LAP were 1.73 ± 1.3 and 58.5 ± 42.3. There were 803 (51.9%) participants with elevated 10-year ASCVD risk. Table 1 presents the baseline characteristics of study participants based on their 10-year ASCVD risk profile. There were obvious differences in age, gender, race, educational level, PIR, smoking status, SBP, DBP, diabetes status, statin use, and aspirin therapy between the two groups (*P* < 0.05). Marital status, BMI, TC, and LDL-C were comparable between the two groups (*P* > 0.05).

**Table 1:** Baseline characteristic of participants.

Variables	Total	Low 10-year ASCVD risk (*n* = 744)	Elevated 10-year ASCVD risk (*n* = 803)	*P*-value
Age, (years)	56.5 ± 10.1	50.0 ± 6.8	62.5 ± 8.9	<0.001
Gender, *n* (%)				<0.001
Male	928 (60.0)	327 (44)	601 (74.8)	
Female	619 (40.0)	417 (56)	202 (25.2)	
Race, *n* (%)				<0.001
White	685 (44.3)	372 (50)	313 (39)	
African American	352 (22.8)	118 (15.9)	234 (29.1)	
Other	510 (33.0)	254 (34.1)	256 (31.9)	
Education level, *n* (%)				<0.001
Did not graduate from high school	329 (21.3)	122 (16.4)	207 (25.8)	
Graduated from high school	379 (24.5)	176 (23.7)	203 (25.3)	
College education or above	839 (54.2)	446 (59.9)	393 (48.9)	
Marital status, *n* (%)				0.147
Married/Living with Partner	967 (62.5)	465 (62.5)	502 (62.5)	
Widowed/Divorced/Separated	423 (27.3)	193 (25.9)	230 (28.6)	
Never married	157 (10.1)	86 (11.6)	71 (8.8)	
PIR	2.6 ± 1.6	2.7 ± 1.7	2.4 ± 1.6	<0.001
Smoking status, *n* (%)				<0.001
Current	594 (38.4)	247 (33.2)	347 (43.2)	
Former	283 (18.3)	135 (18.1)	148 (18.4)	
Never	670 (43.3)	362 (48.7)	308 (38.4)	
BMI, (kg/m^2^)	29.8 ± 6.7	30.0 ± 7.1	29.7 ± 6.4	0.414
SBP, (mmHg)	127.6 ± 16.8	120.5 ± 13.2	134.1 ± 17.2	<0.001
DBP, (mmHg)	75.1 ± 9.5	74.5 ± 8.8	75.7 ± 10.0	0.015
TC, (mg/dl)	196.6 ± 33.1	197.8 ± 32.6	195.5 ± 33.6	0.182
LDL-C, (mg/dl)	118.9 ± 28.9	119.4 ± 27.8	118.5 ± 29.9	0.519
Diabetes, *n* (%)				<0.001
Yes	257 (16.6)	51 (6.9)	206 (25.7)	
Statin use, *n* (%)				<0.001
Yes	363 (23.5)	122 (16.4)	241 (30)	
Aspirin therapy, *n* (%)				<0.001
Yes	432 (27.9)	121 (16.3)	311 (38.7)	
VAI	1.7 ± 1.3	1.5 ± 1.1	1.9 ± 1.4	<0.001
LAP	58.5 ± 42.3	54.3 ± 40.0	62.4 ± 44.0	<0.001

Data were mean ± SD or median (IQR) for skewed variables or numbers (proportions) for categorical variables.

PIR, ratio of family income to poverty; BMI, body mass index; SBP, systolic blood pressure; DBP, diastolic blood pressure; TC, total cholesterol; LDL-C, low density lipoprotein cholesterol; VAI, visceral obesity index; LAP, lipid accumulation product.

### 3.3. Association of VAI and LAP with 10-year ASCVD risk

The univariate analysis demonstrated that age, gender, race, education level, marital status, PIR, smoking status, SBP, DBP, diabetes status, statin use, and aspirin therapy were associated with elevated 10-year ASCVD risk (Supplementary Table S1).

The results of multifactor logistic regression analysis showed that after adjustment in multivariable analyses, VAI and LAP were significantly associated with elevated 10-year ASCVD risk. When VAI was assessed as a continuous variable, the adjusted OR was 3.46 (95% CI: 2.65–4.52) for elevated 10-year ASCVD risk in the full variables adjusted model (model 3). There was a significant positive correlation between VAI and elevated 10-year ASCVD risk after adjusting for all variables, when VAI was analyzed using quartiles. In model 3, compared with participants with the lowest VAI in the 1st Quartile (≤0.79), the adjusted OR values for VAI and elevated 10-year ASCVD risk in Q2 (0.79–1.29), Q3 (1.30–2.14), and Q4 (≥2.15) were 1.50 (95% CI: 0.75–3.00, *P* = 0.254), 2.58 (95% CI: 1.24–5.36, *P* = 0.011), 15.14 (95% CI: 6.93–33.05, *P* < 0.001), respectively (Table 2). When LAP was assessed as a continuous variable, the adjusted OR was 1.04 (95% CI: 1.03–1.05) for elevated 10-year ASCVD risk in model 3. When LAP was analyzed using quartiles, compared with participants with the lowest LAP in the 1st Quartile (≤28.29), the adjusted OR values for VAI and elevated 10-year ASCVD risk in Q2 (28.31–46.44), Q3 (46.52–77.00), and Q4 (≥77.01) were 3.00 (95% CI: 1.49–6.00, *P* = 0.254), 4.63 (95% CI: 2.18–9.82, *P* < 0.001), 16.94 (95% CI: 6.74–42.57, *P* < 0.001), respectively, in model 3 (Table 2). All of the models were statistically significant (Table 2, *P* for trend <0.05).

**Table 2:** Multivariable-adjust ORs and 95% CI of the VAI and LAP quartiles associated with elevated 10-year ASCVD risk.

Variable	Unadjusted		Model 1		Model 2		Model 3	
	OR (95% CI)	*P*-value	OR (95% CI)	*P*-value	OR (95% CI)	*P*-value	OR (95% CI)	*P*-value
VAI	1.25 (1.15 – 1.36)	<0.001	1.95 (1.71 – 2.22)	<0.001	2.25 (1.91 – 2.65)	<0.001	3.46 (2.65 – 4.52)	<0.001
1st Quartile (≤0.79)	1 (Ref)		1 (Ref)		1 (Ref)		1 (Ref)	
2st Quartile (0.79–1.29)	1.35 (1.02 – 1.80)	0.036	1.45 (0.94 – 2.25)	0.095	1.54 (0.92 – 2.59)	0.103	1.50 (0.75 – 3.00)	0.254
3st Quartile (1.30–2.14)	1.70 (1.28 – 2.27)	<0.001	2.64 (1.70 – 4.10)	<0.001	2.73 (1.60 – 4.65)	<0.001	2.58 (1.24 – 5.36)	0.011
4st Quartile (≥2.15)	2.06 (1.54 – 2.74)	<0.001	6.98 (4.41 – 11.05)	<0.001	8.52 (4.90 – 14.81)	<0.001	15.14 (6.93 – 33.05)	<0.001
*P* for trend		<0.001		<0.001		<0.001		<0.001
LAP	1.01 (1.00 – 1.01)	<0.001	1.02 (1.01 – 1.02)	<0.001	1.02 (1.02 – 1.03)	<0.001	1.04 (1.03 – 1.05)	<0.001
1st Quartile (≤28.29)	1 (Ref)		1 (Ref)		1 (Ref)		1 (Ref)	
2st Quartile (28.31–46.44)	1.30 (0.98 – 1.73)	0.067	1.88 (1.22 – 2.90)	0.004	2.37 (1.40 – 4.00)	0.001	3.00 (1.49 – 6.00)	0.254
3st Quartile (46.52–77.00)	1.72 (1.29 – 2.28)	<0.001	2.60 (1.69 – 4.00)	<0.001	3.83 (2.19 – 6.69)	<0.001	4.63 (2.18 – 9.82)	<0.001
4st Quartile (≥77.01)	1.68 (1.27 – 2.23)	<0.001	4.99 (3.21 – 7.76)	<0.001	8.12 (4.38 – 15.05)	<0.001	16.94 (6.74 – 42.57)	<0.001
*P* for trend		<0.001		<0.001		<0.001		<0.001

Model 1 adjust for Age, Gender, Race.

Model 2 adjust for Model 1 + Education level, Marital status, PIR, Smoking status, BMI.

Model 3 adjust for Model 1 + Model 2 + SBP, DBP, TC, LDL-C, Diabetes, Statin use, Aspirin therapy.

Ref, reference; PIR, ratio of family income to poverty; BMI, body mass index; SBP, systolic blood pressure; DBP, diastolic blood pressure; TC, total cholesterol; LDL-C, low density lipoprotein cholesterol; VAI, visceral obesity index; LAP, lipid accumulation product.

**Figure 2:**
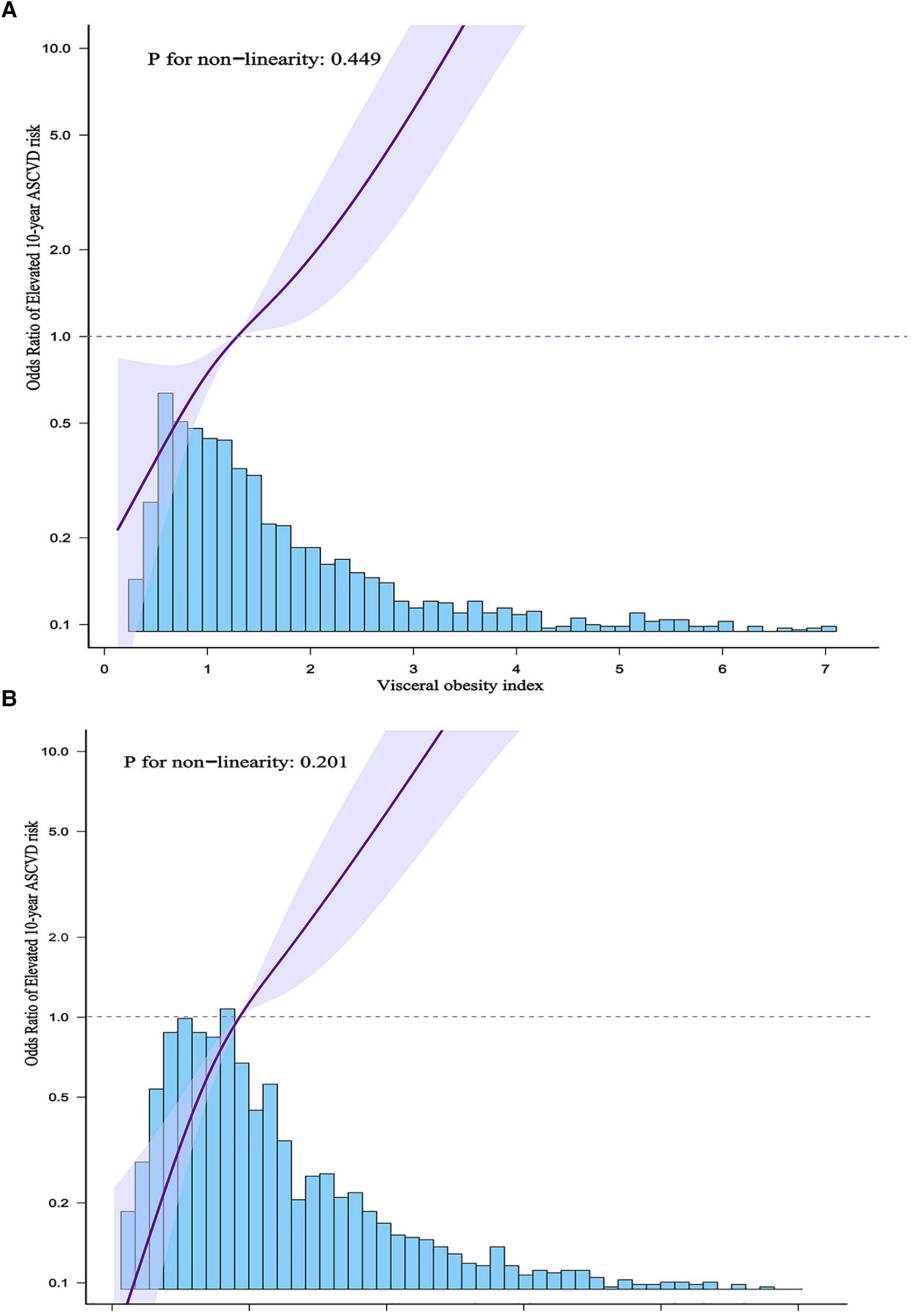
(**A**) Shows the association between VAI and elevated 10-year ASCVD risk. (**B**) Shows the association between LAP and elevated 10-year ASCVD risk. The solid purple line indicates the estimated or predicted value, the shaded area around the solid purple line indicates the 95% confidence interval, and the blue bar provides information on the sample size.

In addition, we used generalized additive models and smoothed curve fittings to assess the links between VAI or LAP and elevated 10-year ASCVD risk (Figure 2). There was a linear relationship of elevated 10-year ASCVD risk with VAI and LAP (*P* for non-linearity >0.05), which indicated that 10-year ASCVD risk increased with VAI and LAP.

### 3.4. Stratified analyses based on additional variables

**Figure 3:**
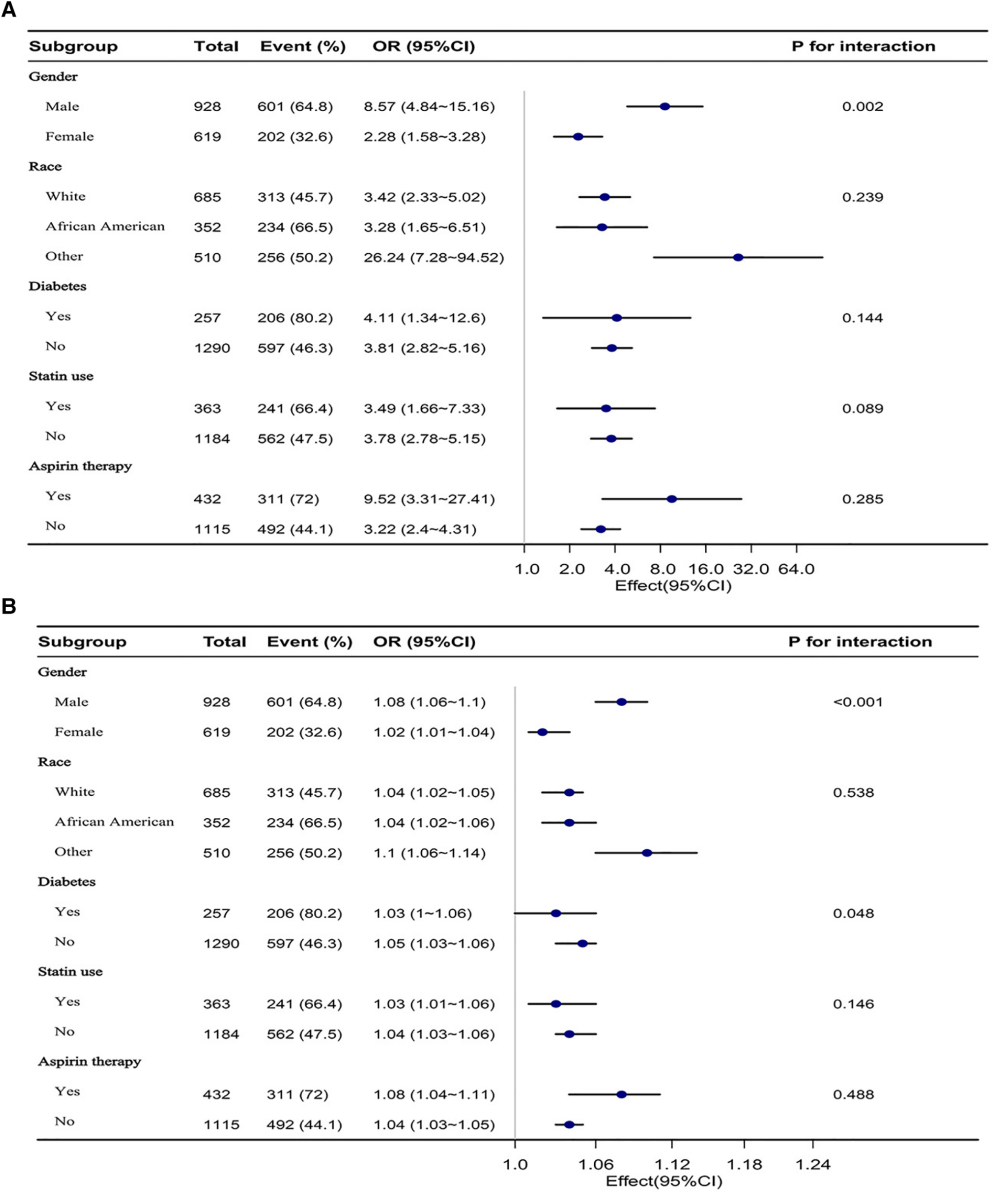
(**A**) Is a forest plot of the subgroup analysis of VAI and elevated 10-year ASCVD risk. (**B**) Is a forest plot of the subgroup analysis of LAP and elevated 10-year ASCVD risk. Each subgroup was adjusted for all other variables except the grouping factor itself.

Stratified analyses were conducted in various subgroups to examine the potential modification effect of VAI and LAP on the relationship with elevated 10-year ASCVD risk (Figure 3). No significant interactions were found in any of the subgroups after stratification by race, diabetes status, statin use, and aspirin therapy (*P* for interaction >0.05). After stratifying by gender, significant interactions were observed in both VAI and LAP groups (*P* for interaction <0.05).

### 3.5. Sensitivity analysis

After excluding participants with extreme VAI and LAP, 1,490 and 1,487 participants were remaining, respectively, and the association between elevated 10-year ASCVD risk with VAI and LAP remained stable. When VAI or LAP was assessed as a continuous variable, in the fully adjusted models, the adjusted OR for a 10-year ASCVD risk increase was 3.04 (95% CI: 2.24–4.12, *P* < 0.001) and 1.03 (95% CI: 1.02–1.04, *P* < 0.001), respectively (Supplementary Table S2).

## 4. Discussion

This is a large cross-sectional study of American adults aged 40–79 years using NHANES data from 2011 to 2020. And the results of the study showed that VAI or LAP, whether as a continuous or categorical variable, was positively and linearly associated with elevated 10-year ASCVD risk when adjusted for potential confounding factors. The relationship between 10-year ASCVD risk with VAI and LAP remained robust after stratified and sensitivity analyses were performed. Interestingly, the stratified analysis also showed that this relationship was more pronounced among men.

It is well known that atherosclerosis is strongly associated with the risk of cardiovascular mortality worldwide (27, 28). Visceral obesity is strongly associated with increased atherosclerotic burden and is an emerging risk factor for CVD (13). And there is research showing that visceral obesity is significantly associated with the risk of recurrent ASCVD after myocardial infarction, residual cardiovascular risk, and CVD mortality (29). What's more a study on the South American population found that visceral obesity accounts for 15.4% of the 12 modifiable risk factors for CVD, ranking second. And its contribution to CVD mortality is 9.7% (30). ASCVD, recurrent cardiovascular events, and residual CVD risks impose a heavy burden on human health and the development of the economy and society. Therefore, paying attention to visceral obesity is crucial for reducing the burden of atherosclerosis. CT, MRI and other imaging methods are the gold standard for detecting visceral obesity, providing a visual display of the thickness and area of visceral fat. However, due to the high cost, time-consuming nature, and the need for professional operators, these imaging examinations are not suitable for large-scale surveys of the general population in clinical settings (31–33). VAI and LAP are considered sensitive and reliable indicators for assessing visceral obesity, especially VAI has been proven to be highly correlated with visceral fat measured through gold standard methods (17, 22). Its advantages of high safety, easy operation, and low cost make it replace complex imaging methods and become an alternative indicator for evaluating visceral obesity. Amato et al. showed for the first time in a retrospective study of Alkam metabolic syndrome (AlkaMeSy) that an increase in VAI was independently associated with increased cardiovascular and cerebrovascular events (18). Subsequent studies suggested that VAI was independently associated with coronary atherosclerosis and could assess cardiometabolic risk, ASCVD risk, and CVD mortality (34–38). However, the relationship between VAI and 10-year ASCVD risk remains controversial. In a prospective cohort study conducted in Europe, Koulii et al. found that VAI was independently associated with a 10-year risk of CVD, especially in males, and its relevance was not affected by potential confounding factors such as lifestyle factors (39). On the contrary, Aysegul et al. conducted a prospective cohort study on 55 postmenopausal women and found that there was no significant association between VAI and 10-year CVD risk (40). Our research on American adults aged 40–79 shows that there is a significant correlation between AVI and the 10-year ASCVD risk, and this relationship is more pronounced in males, which is similar to the findings of Koulii et al. This gender difference may be due to the fact that men and women differ greatly in body fat distribution, with men being more prone to visceral fat accumulation than women (41, 42). And a study showed that the measurement of visceral fat tissue in men using CT scans is twice as high as that in premenopausal women, and postmenopausal women also have lower accumulation of visceral fat tissue. As a result, women have lower risk of cardiovascular metabolic disorders (43, 44). In addition, hormones have a great impact on fat distribution patterns. Research has shown that androgens can promote the accumulation of visceral fat, while estrogens have less impact on the accumulation of visceral fat (43). Therefore, the VAI in males may be relatively higher than in females, with a greater increase in 10-year ASCVD risk. However, whether there are gender differences in the association between VAI and 10-year ASCVD risk still requires further validation through large-scale clinical studies. In 2005, Henry Kahn et al. based on the cross-sectional study of the NHANES III first proposed the LAP index and pointed out that compared with BMI, LAP had a better correlation with key risk factors for CVD (such as heart rate and blood lipids, as well as uric acid circulation levels), and may better predict the incidence of CVD (22). Subsequent studies have shown that LAP was associated with atherosclerosis in elderly and menopausal women, and can independently predict the risk of cardiovascular events in women with polycystic ovary syndrome (PCOS) as well as in participants with normal BMI (35, 44–46). Ioachimescu et al. found that LAP, rather than BMI, can predict the mortality of non-diabetes patients with high CVD risk, which suggested that LAP may be a useful tool for risk stratification of obesity-related adverse consequences in clinical practice (47). Li et al.'s cross-sectional study conducted in China showed that the alternative indicators of visceral obesity, VAI and LAP, may be related to the risk of intracranial Atherosclerosis stenosis (ICAS) in women ≥40 years (48). Kyrou et al. found that LAP was independently related to the long-term incidence of CVD in a prospective study of the Greek population (49), which was similar to our research findings. However, unlike the findings of Huang et al. in 3,143 Taiwanese adults, our study did not find any differences in the association strength between VAI and LAP with 10-year ASCVD risk, which may be due to ethnic differences in the study population (50).

The mechanism by which central obesity indicators (VAI and LAP) are associated with 10-year ASCVD risk is still unclear. There are several possible explanations for the research results. Firstly, the study indicates that abnormal distribution and accumulation of adipose tissue are fundamental causes of atherosclerosis, and VAI and LAP are representative indices for assessing adipose distribution and accumulation (35). Secondly, the characteristic of visceral obesity is an increased deposition of visceral and ectopic fat, which is associated with insulin resistance (51), elevated blood pressure (52), dyslipidemia (53), and inflammation (54), all of which are closely related to ASCVD risk. Visceral adipose tissue can increase basal fat breakdown, release free fatty acids (FFA), and specific cytokines secreted by visceral adipocytes, such as leptin and adiponectin, which can increase insulin resistance (55–57). In addition, inflammatory cytokines (tumor necrosis factor-alpha and interleukin-6) released by macrophages accumulated in visceral adipose tissue can weaken insulin sensitivity and thus promote insulin resistance (58). In insulin resistance, co-causative factors including glucotoxicity, lipotoxicity, and inflammation selectively impair PI3K-dependent insulin signaling pathways, thereby inducing the atherogenic process and leading to the occurrence of ASCVD (59). Hypertension is a recognized risk factor for ASCVD. Visceral adiposity patients have increased insulin and leptin which promotes sympathetic nervous system (SNS) activity (60–62). The SNS stimulates renin release and the production of angiotensin II, which increases the activity of the Renin-Angiotensin-Aldosterone System (RAAS) and therefore raises blood pressure (63, 64). In addition, visceral obesity causes the kidneys to reabsorb sodium through the SNS, hormones (aldosterone and insulin), and renal vasculature (angiotensin II). The increase in sodium also contributes to higher blood pressure to maintain sodium balance and volume homeostasis (65, 66). Dyslipidemia is highly associated with ASCVD risk. Abnormal lipid metabolism causes the blood to be in a highly cohesive state, the blood viscosity increases, and promotes the formation of atherosclerotic plaque (67). Insulin resistance, abnormal metabolism of fat factors [pro-inflammatory adipokines (leptin, resistin, TNF-α), anti-inflammatory adipokine (adiponectin), specific adipokine Sfrp5], and vitamin D deficiency are all possible causes of abnormal blood lipids in visceral obese individuals (53). In addition, excessive production of very low-density lipoprotein (VLDL) by the liver and reduced breakdown of triglycerides (TG) during lipid metabolism circulation, damaged peripheral FFA uptake, increased FFA from adipocytes to the liver and other tissues, and the formation of small dense LDL as well as damage to the ASP/C3adesArg pathway are also possible mechanisms of obesity-induced abnormal blood lipids (68). Inflammation is a key link in the occurrence and development of ASCVD. When there is excessive visceral fat, subcutaneous enlarged adipocytes secrete pro-inflammatory cytokines such as IL-6, reducing the secretion of possible anti-inflammatory and insulin sensitized cytokines adiponectin, and prone to cell apoptosis, leading to macrophage invasion (69–71). Macrophages infiltrate into enlarged adipocytes, further leading to an increase in the production of inflammatory cytokines such as tumor necrosis factor-alpha and interleukin-6, a decrease in the production of protective adipokine adiponectin, and harmful cross-talk between macrophages and enlarged adipocytes resulting in the production of detrimental secreted products (13, 72).

To our knowledge, this is the first exploration of the relationship between VAI and LAP with 10-year risk of first ASCVD events in US adults. However, there also are some limitations in our study. Firstly, although regression models, subgroup analysis, and sensitivity analysis are used, residual confounding effects of unmeasured or unknown factors cannot be completely excluded. Secondly, the current research results are based on a survey of adults aged 40–79 in the United States, and further research is still needed to determine whether the results of this study are applicable to other populations. In addition, although one of the indicators of visceral obesity, VAI, is a composite calculated from BMI, WC, TG and HDL-C. However, it has a similar parameter to the ASCVD, and some validation bias may exist even though the primary results did not change after adjusting for the similar parameter. Finally, the cross-sectional study can only explore the correlation, and can not further draw causal inferences, thus future longitudinal studies or randomized controlled trials are needed for further validation.

## 5. Conclusion

In conclusion, in American adults, especially males, VAI or LAP score is positively correlated with 10-year risk of first ASCVD events. Our research indicates that doctors should assess the degree of visceral obesity to identify individuals at high risk for ASCVD**.**

## Data Availability

Publicly available datasets were analyzed in this study. This data can be found here: www.cdc.gov/nchs/nhanes/.
