# Supplementary material for: Association between visceral obesity and 10-year risk of first atherosclerotic cardiovascular diseases events among American adults: National Health and Nutrition Examination Survey
Source: Front Cardiovasc Med. 2023 Aug 21;10:1249401. doi: 10.3389/fcvm.2023.1249401 (PMC10479018; doi:10.3389/fcvm.2023.1249401)
Supplement: Supplementary file 1 [file Table1.doc]

**Table S1 | Results of univariate analysis of elevated 10-year ASCVD risk.**

| **Variable** | **OR (95%CI)** | **P-value** |
| --- | --- | --- |
| Age, (years) | 1.2 (1.18~1.22) | <0.001 |
| Gender, n (%) |  |  |
| Male | Ref |  |
| Female | 0.26 (0.21~0.33) | <0.001 |
| Race, n (%) |  |  |
| White | Ref |  |
| African American | 2.36 (1.8~3.08) | <0.001 |
| Other | 1.2 (0.95~1.51) | 0.123 |
| Education level, n (%) |  |  |
| Did not graduate from high school | Ref |  |
| Graduated from high school | 0.68 (0.5~0.92) | 0.012 |
| College education or above | 0.52 (0.4~0.67) | <0.001 |
| Marital status, n (%) |  |  |
| Married/Living with Partner | Ref |  |
| Widowed/Divorced/Separated | 1.1 (0.88~1.39) | 0.398 |
| Never married | 0.76 (0.55~1.07) | 0.121 |
| PIR | 0.9 (0.84~0.95) | 0.001 |
| Smoking status, n (%) |  |  |
| Current | Ref |  |
| Former | 0.78 (0.59~1.04) | 0.088 |
| Never | 0.61 (0.48~0.76) | <0.001 |
| BMI, (kg/m2) | 0.99 (0.98~1.01) | 0.413 |
| SBP, (mmHg) | 1.06 (1.05~1.07) | <0.001 |
| DBP, (mmHg) | 1.01 (1~1.02) | 0.015 |
| TC, (mg/dL) | 1 (0.99~1) | 0.182 |
| LDL-C, (mg/dL) | 1 (1~1) | 0.518 |
| Diabetes |  |  |
| Yes | Ref |  |
| No | 0.21 (0.15~0.3) | <0.001 |
| Statin use, n (%) |  |  |
| Yes | Ref |  |
| No | 0.46 (0.36~0.58) | <0.001 |
| Aspirin therapy, n (%) | Ref |  |
| Yes |  |  |
| No | 0.31 (0.24~0.39) | <0.001 |
| VAI | 1.25 (1.15~1.36) | <0.001 |
| LAP | 1 (1~1.01) | <0.001 |

Ref, reference; PIR, ratio of family income to poverty; BMI, body mass index; SBP, systolic blood pressure; DBP, diastolic blood pressure; TC, total cholesterol; LDL-C, low density lipoprotein cholesterol; VAI, visceral obesity index; LAP, lipid accumulation product.
