# Supplementary material for: Association between visceral obesity and 10-year risk of first atherosclerotic cardiovascular diseases events among American adults: National Health and Nutrition Examination Survey
Source: Front Cardiovasc Med. 2023 Aug 21;10:1249401. doi: 10.3389/fcvm.2023.1249401 (PMC10479018; doi:10.3389/fcvm.2023.1249401)
Supplement: Supplementary file 2 [file Table2.doc]

**Table S2 |** Multivariable-adjust ORs and 95%CI of the VAI and LAP quartiles associated with elevated 10-year ASCVD risk. Extreme VAI and LAP were not included.

| **Variable** | **Unadjusted** | |  | **Model 1** | |  | **Model 2** | |  | **Model 3** | |
| --- | --- | --- | --- | --- | --- | --- | --- | --- | --- | --- | --- |
|  | **OR (95%CI)** | **P-value** |  | **OR (95%CI)** | **P-value** |  | **OR (95%CI)** | **P-value** |  | **OR (95%CI)** | **P-value** |
| VAI | 1.23 (1.11~1.37) | <0.001 |  | 1.86 (1.58~2.19) | <0.001 |  | 1.98 (1.63~2.41) | <0.001 |  | 3.04 (2.24~4.12) | <0.001 |
| 1st Quartile (≤0.77) | 1(Ref) |  |  | 1(Ref) |  |  | 1(Ref) |  |  | 1(Ref) |  |
| 2st Quartile (0.78-1.24) | 1.34 (1.00~1.80) | 0.046 |  | 1.55 (0.98~2.45) | 0.058 |  | 1.68 (0.97~2.9) | 0.063 |  | 1.74 (0.83~3.65) | 0.143 |
| 3st Quartile (1.25-2.01) | 1.76 (1.32~2.35) | <0.001 |  | 2.91 (1.85~4.57) | <0.001 |  | 2.98 (1.72~5.15) | <0.001 |  | 2.62 (1.23~5.57) | 0.012 |
| 4st Quartile (2.02-5.07) | 1.75 (1.31~2.34) | <0.001 |  | 4.7 (2.96~7.45) | <0.001 |  | 5.44 (3.13~9.45) | <0.001 |  | 10.19 (4.64~22.36) | <0.001 |
| P for trend |  | <0.001 |  |  | <0.001 |  |  | <0.001 |  |  | <0.001 |
| LAP | 1.00 (1.00~1.01) | 0.026 |  | 1.01 (1.01~1.02) | <0.001 |  | 1.02 (1.01~1.03) | <0.001 |  | 1.03 (1.02~1.04) | <0.001 |
| 1st Quartile (≤27.69) | 1(Ref) |  |  | 1(Ref) |  |  | 1(Ref) |  |  | 1(Ref) |  |
| 2st Quartile (27.70-44.92) | 1.39 (1.04~1.85) | 0.026 |  | 1.86 (1.18~2.92) | 0.008 |  | 2.34 (1.35~4.07) | 0.003 |  | 3.25 (1.53~6.87) | 0.002 |
| 3st Quartile (45.03-72.79) | 1.62 (1.21~2.16) | 0.001 |  | 2.55 (1.62~4.02) | <0.001 |  | 3.5 (1.93~6.36) | <0.001 |  | 5.3 (2.34~11.98) | <0.001 |
| 4st Quartile (≥72.96) | 1.57 (1.18~2.1) | 0.002 |  | 3.57 (2.27~5.61) | <0.001 |  | 5.55 (2.92~10.55) | <0.001 |  | 15.31 (5.66~41.37) | <0.001 |
| P for trend |  | 0.001 |  |  | <0.001 |  |  | <0.001 |  |  | <0.001 |

Model 1 adjust for Age, Gender, Race.

Model 2 adjust for Model 1+Education level, Marital status, PIR, Smoking status, BMI.

Model 3 adjust for Model 1+Model 2+SBP, DBP, TC, LDL-C, Diabetes, Statin use, Aspirin therapy.

Ref, reference; PIR, ratio of family income to poverty; BMI, body mass index; SBP, systolic blood pressure; DBP, diastolic blood pressure;

TC, total cholesterol; LDL-C, low density lipoprotein cholesterol; VAI, visceral obesity index; LAP, lipid accumulation product.
